# Development, Implementation, and Evaluation of a Prevention Model of Care in South West Queensland (Wellness my Way): Protocol for a Pilot Implementation Study

**DOI:** 10.2196/98966

**Published:** 2026-09-03

**Authors:** Claudia I Maddren, Clare Johnson, Tahlia Alsop, Madeline Forbes, Li Kheng Chai, Joanne Isbel, Kathy Morrow, Joanna Munro, Clare Murray, Shelley Peardon-Freeman, Claudia Regan-Knights, Helen Wassman, Sjaan R Gomersall

**Affiliations:** 1 Health and Wellbeing Centre for Research Innovation, School of Human Movement and Nutrition Sciences, The University of Queensland, St. Lucia, Queensland, 4072, Australia, 61 07-3365-1111; 2 Health and Wellbeing Queensland, Milton, Queensland, Australia; 3 Health Contact Centre, Clinical Excellence Queensland, Queensland Health, Brisbane, Queensland, Australia; 4 South West Hospital and Health Service, Queensland Health, Roma, Queensland, Australia; 5 School of Health and Rehabilitation Sciences, The University of Queensland, St Lucia, Queensland, Australia

**Keywords:** prevention, health promotion, digital health, chronic disease, evaluation

## Abstract

**Background:**

Wellness my Way is a chronic disease prevention model of care being piloted across South West Queensland, Australia, a region of high chronic disease burden. Wellness my Way is an initiative of the Queensland Government through Health and Wellbeing Queensland, delivered in partnership with Queensland Health’s Health Contact Centre and South West Hospital and Health Service, supported by multisectoral collaborations.

**Objective:**

This protocol aims to describe the development of Wellness my Way and the protocol for evaluating the program’s pilot implementation.

**Methods:**

The context for the Wellness my Way pilot and the multisectoral approach and co-design activities that informed program development and implementation are described. The RE-AIM (Reach, Effectiveness, Adoption, Implementation, and Maintenance) framework has guided the evaluation. The primary outcome is the uptake (reach) of Wellness my Way. Secondary outcomes include consumer satisfaction and stakeholder experiences. Data were derived from quantitative and qualitative measures collected from consumers and stakeholders.

**Results:**

Ethics approval has been granted. The Wellness my Way pilot program within South West Queensland, Australia, concluded in January 2026, with findings anticipated to be shared in late 2026.

**Conclusions:**

The findings will offer new insights on the impact of a collaboratively delivered program in a community with high unmet needs.

## Introduction

Noncommunicable diseases (NCDs) are complex health conditions that can lead to lower quality of life and shorter life expectancy [1]. Many NCDs are linked to modifiable risk factors such as consumption of a poor diet, physical inactivity, overweight and obesity, smoking, and harmful drug and alcohol use [1,2]. In Australia, 1 in 2 people are living with at least 1 NCD [3]. In 2023‐2024, NCDs accounted for two-thirds of the total burden of disease, 52% of hospitalizations, and 89% of deaths, and contributed to AUD $82 billion (US $58 billion) in health care expenditure [4-6]. For regional and rural Australians, higher rates of NCDs are experienced with 67% of these residents living with at least 1 condition compared to 58% of metropolitan residents [7]. Australians living in these areas disproportionately report higher rates of behaviors associated with poorer health outcomes, compared to those living in major cities, influenced by barriers in health infrastructure and the provision of health care services and prevention [7,8].

The 2022 Australian National Health Survey reported 52% of residents in the Australian state of Queensland were living with at least 1 NCD [8]. Most recent estimations from 2011 to 2012 found that health expenditure in Queensland associated with NCDs was estimated at AUD $9.6 billion (US $6.8 billion) [9]. Fragmented health care systems, time and financial constraints, and workforce shortages have created barriers to prevention efforts [10]. Limited community knowledge of prevention, lack of awareness of NCDs, and low engagement in behavior change have also been reported as barriers [11].

A range of free and low-cost prevention programs are available to Queenslanders; however, gaps in awareness, health literacy, geographical barriers, and complex service pathways have reportedly contributed to poor uptake [12]. To address this, a prevention model of care, Wellness my Way [13], was designed, led, and funded by Health and Wellbeing Queensland (HWQld), in partnership with Queensland Health’s Health Contact Centre (HCC), Queensland Health’s South West Hospital and Health Service’s (SWHHS) Healthy Communities Team, and the Health and Wellbeing Centre for Research Innovation (HWCRI) at The University of Queensland (UQ). Wellness my Way is a free, personalized approach to prevention piloted in South West Queensland [13]. The program seeks to increase the uptake of prevention programs through a community-led, streamlined approach, including an online health and well-being assessment and telephone-delivered coaching to co-design a personal well-being action plan. This program leverages a pre-existing prehabilitation model of care delivered by HCC, Way to Wellness.

Health coaching is an evidence-informed approach to health promotion with digital health improving reach and scalability [14,15]. The program uses a place-based approach to build preventive health capacity in local workforces, integrate prevention into existing systems, and connect consumers to existing prevention programs and services [16].

The objective of this study is to describe the development of, and the evaluation protocol for, Wellness my Way. We outline the program’s context and setting, and describe the multisectoral approach and co-design activities which led to the program’s development, recruitment, and delivery. The detailed evaluation protocol is also described.

## Methods

The Template for Intervention Description and Replication–Population Health and Policy (TIDieR-PHP) interventions [17] and Expert Recommendations for Implementing Change (ERIC) strategies [18] were used to guide the reporting of this protocol. The Wellness my Way pilot was delivered from July 29, 2024, to June 30, 2025, with follow-up data collection until January 29, 2026.

### Context and Setting

The Wellness my Way pilot was conducted in the South West region of Queensland, in Queensland, Australia. In 2022, South West Queensland had a population of 24,173 (50% female), and 56% were aged between 20‐64 years [19]. In 2025, 16% of South West Queensland identified as First Nations [20]. Recent data estimate that South West Queensland adults experience high rates of modifiable risk factors, with just over two-thirds living with overweight or obesity, and almost 50% do not engage in sufficient levels of physical activity [21]. Over 90% reported inadequate consumption of vegetables, 27% reported risky alcohol use, and 13% reported smoking daily [21].

### Program Development

In 2022, HWQld, Queensland Government’s statutory agency for chronic disease prevention, set out to improve awareness, navigation, and uptake of prevention programs. Stakeholder consultation explored both clinician- and consumer-facing solutions, identifying an opportunity to work collaboratively with the health system to test a free, consumer-facing digital pathway to a prevention program in Queensland leveraging the HCC Way to Wellness prehabilitation model of care. Key elements of Way to Wellness include SMS text message invitation, online health assessment to identify modifiable risk factors, provision of brief advice in line with Australian clinical guidelines, telephone coaching using motivational interviewing to develop a wellness action plan delivered by a preventive health counselor, promotion of the role of general practitioners in supporting the consumer to make health changes (including sharing of the wellness action plan), and offer of connecting consumers with prevention programs while awaiting orthopedic surgery [22]. HWQld and HCC collaboratively adapted the Way to Wellness model to be accessible for more Queenslanders to support early identification and reduce modifiable risk factors for those at risk for, in the early stages of, or living with NCDs [23].

South West Queensland, a region with high burden of NCDs, was considered an appropriate setting for the Wellness my Way pilot, with implementation supported by an existing memorandum of understanding and strategic alignment between HWQld and SWHHS. The SWHHS’ Healthy Communities Team were engaged as the local implementation partner, bringing knowledge of the regional context and guiding engagement with the community.

Subsequently, HWQld led the development of a place-based implementation plan focused on collaboration, community activation, building workforce capability, and shared responsibility. This was achieved through in-person and online consultations with SWHHS’ Community Advisory Networks and local stakeholders to gather feedback on the model of care, identify local referral pathways, and inform implementation opportunities, including the design of a localized marketing campaign.

In 2023, leveraging an established partnership with UQ through a jointly funded research center by HWQld and UQ, HWQld partnered with HWCRI for expert consultation on health behavior change, research, and leading the evaluation of the Wellness my Way pilot.

The development phase culminated in a multisectoral partnership, led and funded by HWQld, that included Queensland Health’s SWHHS Healthy Communities Team for local implementation, Queensland Health’s HCC for delivery of the model of care, and HWCRI at UQ for research and evaluation.

### Model of Care

Wellness my Way involves 3 primary components. First, Wellness my Way invites consumers to complete a health and well-being assessment online or via telephone to assess current health status [13]. Using validated tools [24-26], questions explore the consumer’s self-reported health behaviors, including nutrition, physical activity level, mental health status, smoking, and drug and alcohol use; biometrics such as height, weight, and blood glucose level history; and demographics. A summary of the health and well-being assessment is emailed to the consumer. Next, within 1 week of the assessment, a preventative health counselor from the HCC contacts the consumer. Applying motivational interviewing skills [27], the preventative health counselor works with the consumer to co-design individual goals and create a well-being action plan, and provide referrals to free or low-cost, locally accessible prevention programs and services. The well-being action plan is shared with the consumer and, upon consent, shared with their nominated primary care professional. The consumer then continues their health journey with the support of the identified prevention programs or pathways.

Prevention programs available to consumers include a range of free and low-cost programs delivered locally (eg, SWHHS Community Dietitian, SWHHS Exercise Physiology, Parkrun), or at a state or national level (eg, Quitline, My health for life), all focused on supporting health behavior change. Programs may be delivered online, via phone, or in person. Consumers can be directly referred into programs with established referral pathways (upon consent) or provided program information for self-enrollment.

### Consumer Eligibility

Wellness my Way is available to individuals 18 years and older, and is most suited for those at risk of developing or living with NCDs. Pregnant people or those younger than 18 years are not eligible to participate. While not exclusively available to people living in South West Queensland, pilot recruitment is intentionally targeted toward residents living in this region.

### Recruitment

Consumers were recruited using a range of strategies, including community activation, local marketing campaigns, and waitlists. Recruitment via specialist outpatient waitlists was undertaken by HCC, consistent with the existing prehabilitation model of care. Eligible patients on South West Queensland category 2 (semiurgent specialist consultation) and 3 (nonurgent specialist consultation) waitlists received an SMS text message invitation linking to the online health and well-being assessment.

### Implementation Strategies

The Wellness my Way pilot program used a range of implementation strategies described in detail below and summarized in Table 1, where the primary implementation strategies are mapped according to Powell et al’s [18] ERIC strategies.

**Table 1.** Wellness my Way implementation activities mapped to Expert Recommendations for Implementing Change (ERIC) strategies.

ERIC strategies	Connectors	Learning workshops	Marketing	Resources	Partnerships^a^
Access new funding					✓
Assess for readiness and identify barriers and facilitators					✓
Audit and provide feedback		✓			✓
Build a coalition	✓	✓			✓
Capture and share local knowledge	✓	✓			✓
Conduct cyclical small tests of change	✓	✓			
Conduct educational meetings	✓	✓			✓
Conduct educational outreach visits	✓	✓			✓
Conduct local consensus discussions	✓	✓			
Conduct local needs assessment					✓
Conduct ongoing training		✓			
Create a learning collaborative	✓	✓			✓
Develop a formal implementation blueprint				✓	✓
Develop academic partnerships		✓			✓
Develop an implementation glossary				✓	
Develop and implement tools for quality monitoring					✓
Develop educational materials			✓	✓	✓
Develop resource sharing agreements					✓
Distribute educational materials	✓	✓			✓
Facilitate relay of clinical data to providers	✓				✓
Facilitation	✓	✓			✓
Fund and contract for the clinical innovation					✓
Identify and prepare champions	✓	✓	✓		
Identify early adopters	✓	✓			
Increase demand	✓		✓		
Inform local opinion leaders	✓	✓			✓
Intervene with patients/consumers to enhance uptake and adherence	✓	✓			
Involve executive boards					✓
Involve patients/consumers and family members	✓		✓		
Make training dynamic	✓	✓			
Mandate change					✓
Model and stimulate change					✓
Obtain and use patients/consumers and family feedback	✓				✓
Obtain formal commitments	✓				✓
Organize clinician implementation team meetings	✓	✓			✓
Promote adaptability	✓	✓			✓
Promote network weaving	✓	✓			✓
Provide local technical assistance	✓	✓		✓	✓
Provide ongoing consultation		✓			✓
Purposely reexamine the implementation		✓			✓
Recruit, designate, and train for leadership					✓
Remind clinicians	✓	✓	✓	✓	✓
Revise professional roles	✓				
Shadow other experts	✓	✓			✓
Stage implementation scale-up	✓	✓	✓	✓	✓
Start a dissemination organization					✓
Tailor strategies	✓	✓	✓	✓	✓
Use advisory boards and workgroups	✓	✓			✓
Use an implementation advisor					✓
Use data experts					✓
Use data warehousing techniques					✓
Use mass media	✓		✓		
Use train-the-trainer strategies	✓				

a Partnerships describe the collective work between Health and Wellbeing Queensland, Queensland Health’s South West Hospital and Health Service, Queensland Health’s Health Contact Centre, and Health and Wellbeing Centre for Research Innovation. Only relevant strategies are included in this table.

### Training of Preventive Health Counselors

Prior to the pilot launch, HWQld and SWHHS delivered and recorded an online training session to upskill HCC preventive health counselors. This included aims and objectives of Wellness my Way, and regional needs of those living in South West Queensland, including geographic, social, and contextual factors such as limited transport, geographical distances, and harsh climates. Ongoing training was facilitated by local program leads (eg, community dietitian, mental health professionals) to inform counselors of regional services.

### Connectors

HWQld and SWHHS identified, supported, and upskilled local “Connectors”—local voices who advocate for, raise awareness of, and encourage participation in Wellness my Way. This approach was designed to make prevention a shared responsibility across the community, creating multiple entry points into Wellness my Way and prevention programs [28]. Connectors could be situated in settings such as local government, hospitals, pharmacies, allied health, general practices, Aboriginal and Torres Strait Islander Controlled Health Organizations, workplaces, nongovernment organizations, local businesses, community centers, and support groups.

HWQld invited South West community and social service organizations and health services to submit an Expression of Interest to assist implementation and embed Wellness my Way within their current service delivery. Each participating organization established a team of Connectors responsible for actioning Wellness my Way within their own organization. HWQld and SWHHS supported interested organizations and their identified Connectors via informal online check-ins to support continuous quality improvement, mitigate any issues, and drive motivation.

### Marketing Campaign

HWQld led the design and delivery of a locally tailored marketing campaign across South West Queensland during the pilot. Informed through community consultation, locally representative messaging that featured local faces and places were delivered through print and paid digital media channels to reach people in everyday settings and encourage completion of the health and well-being assessment. Physical materials (eg, recipe cards, flyers, posters, stickers, and coasters) included a QR code. Billboards were displayed on main roads throughout the region. Materials were disseminated by Connectors and the local implementation team at community events, health services, community meeting places, local businesses, and online.

### Learning Workshops

**Figure 1.**
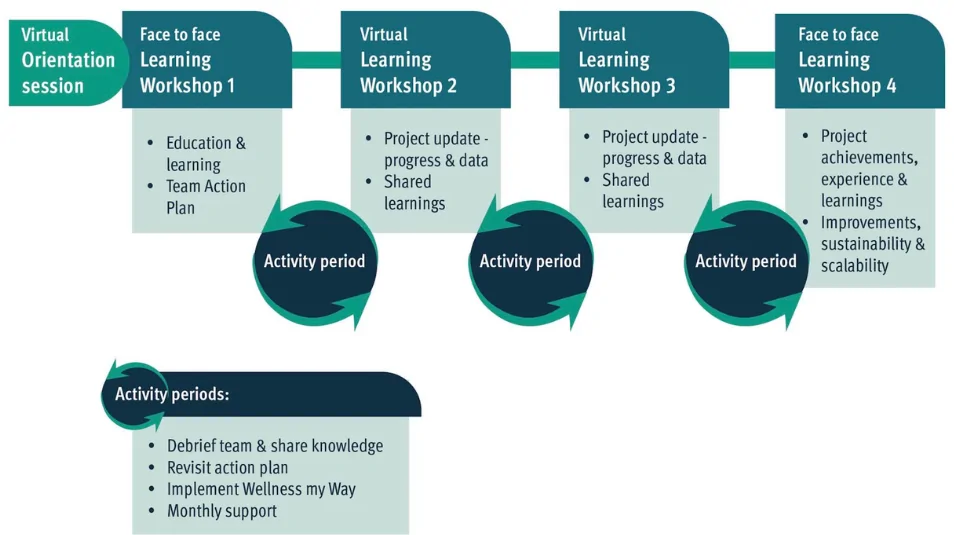
Wellness my Way collaborative methodology implementation strategy (Queensland Government, Health and Wellbeing Queensland, 2025).

Led by HWQld, Connectors were invited to attend 4 learning workshops during the pilot to reflect on data, share learnings, receive support from HWQld and SWHHS, and identify and test avenues to better integrate the program within their organization (Figure 1). Learning workshops were informed by an adapted collaborative methodology [29], which supports shared, community-led action and continuous quality improvements to embed the program into local implementation systems.

### Resources to Support Local Implementation

Informed by a desktop review, HWQld designed specific resources to support Connectors to effectively plan and implement change throughout the pilot. The Implementation Toolkit was developed to support organizations participating in the learning workshops with application of continuous quality improvement principles and testing of implementation approaches within their organizational and community contexts. It includes a Team Action Plan, an adapted version of The Model for Improvement [30], and supports identification of context-specific implementation approaches for iterative testing during the pilot.

The Connector’s guide is an abridged version of the Implementation Toolkit designed for a broader range of stakeholders acting as Connectors across the region, providing an overview of the prevention model of care and guidance to promote Wellness my Way within their communities. Both resources contain a list of prevention pathways on offer and guidance on how to have proactive and supportive conversations with prospective consumers [23]. HWQld has also designed a dedicated website [13], which features further resources to assist pilot implementation.

### Evaluation Protocol for Wellness My Way Pilot Prevention Model of Care

Wellness my Way is a health promotion program delivered by the Queensland Government. A comprehensive evaluation protocol was collaboratively designed by HWQld and HWCRI to examine program uptake, effectiveness, implementation experiences, and sustainability. All data that will be used in the evaluation were collected as part of routine service delivery by Queensland Health and HWQld, with data used for research purposes by UQ.

### Study Design

The evaluation will use a mixed methods approach, guided by the RE-AIM (Reach, Effectiveness, Adoption, Implementation, and Maintenance) framework [31]. Effectiveness will be assessed using a pragmatic single-arm, pre-post study design.

### Objectives

The evaluation of the Wellness my Way pilot aims to address 9 research objectives (Table 2). The primary objective is uptake (reach) of Wellness my Way (research objective 1) with secondary objectives evaluating program effectiveness, adoption, and implementation experiences.

**Table 2.** Wellness my Way pilot evaluation research objectives.

Research objective	RE-AIM^a^ principle
1. What is the reach of Wellness my Way?	Reach
2. What was the uptake of prevention programs as a result of Wellness my Way?	Effectiveness
3. What is the impact of Wellness my Way on chronic disease risk factors of consumers?	Effectiveness
4. How many local organizations partnered with Wellness my Way for implementation in South West Queensland?	Adoption
5. What was the fidelity of the intervention delivery?	Implementation
6. How satisfied were consumers with Wellness my Way and their experience as a participant?	Implementation
7. What are the participants’ perceptions of their experience participating in Wellness my Way?	Implementation
8. What are the community stakeholders’ perceptions of their experience participating in Wellness my Way?	Implementation
9. What are the preventive health counselors perceptions of delivering Wellness my Way?	Implementation

a RE-AIM: Reach, Effectiveness, Adoption, Implementation, and Maintenance.

### Measures

#### Reach

Engagement with Wellness my Way was described as the number of consumers who initiate and complete a health and well-being assessment, engage with telephone coaching to co-design a well-being action plan, and accept referrals to prevention programs. These outcomes were analyzed using data from Queensland Health’s HCC’s Microsoft Dynamics 365 customer relations management (CRM).

#### Effectiveness

##### Uptake of Prevention Programs

The impact of Wellness my Way on participation in prevention programs in South West Queensland was assessed by examining the number of referrals that consumers action. Overall engagement of South West Queensland consumers in prevention services (programs directly or indirectly embedded within Wellness my Way) will be analyzed. Prevention programs were asked to report total program engagement before and during the implementation of the Wellness my Way pilot.

##### Chronic Disease Risk Factors

The impact of Wellness my Way on consumers’ chronic disease–related risk factors and diagnosis was assessed at program intake via the health and well-being assessment and 6 months post engagement with Wellness my Way via a digital or phone survey. Data were collected by Queensland Health’s HCC’s Microsoft Dynamics 365 CRM. Both the health and well-being assessment and the 6-month survey measured diabetes risk using a modified version of the Australian Diabetes Risk Assessment Tool (AUDRISK) [24], mental health using an ultra-brief screening tool (4-item Patient Health Questionnaire [PHQ-4]) [25], physical activity engagement using The Active Australia Survey [26], and other modifiable risk factors such as drug and alcohol use and biometrics.

##### Adoption

Adoption was measured by reporting the number of local organizations that partner with Wellness my Way to support the community-led implementation of the program. This outcome was reported using data collected by Health and Wellbeing Queensland.

### Implementation

#### Fidelity

Intervention fidelity was evaluated by reporting the completion rate for the components of the model of care (health and well-being assessment, telephone coaching). These outcomes were analyzed using data from Queensland Health’s HCC’s Microsoft Dynamics 365 CRM.

#### Consumers

Consumers’ satisfaction and experience with the Wellness my Way pilot were collected via the Patient Reported Experience Measures survey following the health and well-being assessment or telephone coaching call. The survey was sent to consumers who completed the model of care and those who drop out after the health and well-being assessment. Consumers were asked to provide feedback on the prevention model of care via level of agreement using Likert scale items and open text responses.

#### Stakeholders

Stakeholders’ perceptions and experiences of participating in the Wellness my Way pilot were evaluated via a qualitative in-person workshop, semistructured interviews, and focus groups. Program stakeholders including Connectors and HCC preventive health counselors were invited to offer their experience during the pilot implementation.

### Statistical Analysis

All quantitative reach, effectiveness, adoption, and implementation outcomes will be described using descriptive statistics. *χ*^2^ tests of association will be used to examine associations between categorical variables, and paired independent *t* tests and McNemar tests will be used to examine changes in continuous and categorical outcomes, respectively, between program intake and at 6 months. To be eligible to be included in the quantitative evaluation, participants must have been eligible for their 6-month follow-up at the close of the pilot implementation phase. Intention-to-treat analysis will be followed to handle missing data. The effectiveness outcome will be treated as exploratory given the pilot study design and absence of randomization or a comparison group.

All qualitative implementation data will be analyzed according to Braun and Clarke’s [32] approach to thematic analysis. Audio-recorded qualitative data will be transcribed verbatim, checked for accuracy, and deidentified for anonymity. Using NVivo 14 (version 14.3.2; Lumivero), researchers will generate initial codes and meet to group codes into relevant themes. Themes will be refined, and subthemes may be generated to assist interpretation of findings. Open-ended responses will be analyzed using content analysis [33].

### Ethical Considerations

Wellness my Way is a Queensland Government–funded health service program delivered collaboratively by HWQld and Queensland Health. All aspects of program delivery are routine service delivery, and all data collected from consumers were routinely collected data as part of that routine service delivery. At program enrollment, all consumers provided consent for their data to be collected and used for program delivery, evaluation, and future research purposes. Consumer data were routinely collected and securely stored within Queensland Health systems from July 2024 as part of standard program operations. Ethical approval to access these routinely collected data for research purposes was provided by the Metro North Health Human Research Ethics Committee (HREC/2025/MNH/121442; SSA/2025/QHC/121442) on December 24, 2025. Deidentified consumer data will be transferred to HWQld and UQ upon completion of the pilot.

Ethics approval for all qualitative research activities with stakeholders, including workshops, focus groups, and interviews, was obtained from UQ Human Research Ethics Committee (2025/HE000769) on June 13, 2025. All participants in stakeholder consultation were asked to provide prospective informed consent for data collected during consultation to be used for research purposes. Stakeholders who did not provide informed consent were still able to participate in consultation, without their data being used for research purposes.

Data sharing between HWQld, Queensland Health, and UQ was undertaken in accordance with approved ethics, governance, and data sharing agreements. Quantitative consumer data are stored in Queensland Health systems, and only authorized, ethics-approved researchers have access to deidentified data for research purposes upon completion of the pilot. Qualitative data are stored within UQ’s Research Data Manager and only accessible to authorized, ethics-approved researchers for analysis and reporting, in accordance with institutional and national research ethics requirements.

## Results

The Wellness my Way pilot evaluation concluded on January 29, 2026, with results expected to be shared in the second half of 2026. Findings from both quantitative and qualitative analyses will be disseminated in internal reports, conference abstracts, and scientific publications (with authorship determined according to scientific authorship guidelines). Findings will be shared with the pilot’s Connectors as well as consumers via websites, social media, online newsletters, and written reports.

## Discussion

### Principal Findings

This program evaluation will provide key insights to determine whether Wellness my Way has connected people at risk of or living with noncommunicable diseases with free and low-cost prevention programs across South West Queensland. It is expected that Wellness my Way will improve uptake of local and state prevention programs among the pilot population. Findings will inform ongoing program delivery and provide insights into program scalability.

### Comparison to Prior Work and Contribution to the Evidence Base

The Wellness my Way pilot evaluation will occur in collaboration between government entities and university partners, enabling evidence-informed strategic planning across multisectoral partners to strengthen systems and services with the capacity to deliver Wellness my Way at scale. This collaboration is key to generating practice-based evidence, improving the understanding of the impact of community-led prevention programs, and addressing the knowledge-to-practice gap.

National and state governments have called for multisector collaboration to meet the high demands of the health care system [12], particularly in response to the growing burden of chronic disease. Similar work [34] has illustrated how multisector partnerships can codeliver and evaluate a community-delivered health promotion intervention; however, they are often not reported in the scientific literature nor supported by robust evaluation. The current approach ensures that these important and novel approaches to community health promotion are well-documented, described, and evaluated, so that learnings can be disseminated and inform other initiatives, as well as scale up.

### Strengths and Limitations

A strength of the development, implementation, and evaluation protocol for the Wellness my Way pilot is the multisectoral partners across health, government, and academia to design and codeliver the prevention model of care. The evaluation is guided by components of the RE-AIM implementation framework [31], with data collection embedded within program delivery. Reliance on self-report data collected via the health and well-being assessment (eg, chronic disease risk factors) and program engagement data (varied data availability) may introduce response bias. The pragmatic nature of evaluating routine service delivery also means that a single-arm, pre-post study design was used, with more rigorous methods such as those with comparison groups or randomization not feasible.

### Future Directions and Dissemination

Findings from the Wellness my Way pilot evaluation in South West Queensland will inform the potential for scale-up. Dissemination will include peer-reviewed publications, conference presentations, government reports, and plain language summaries for community partners.

### Conclusions

The development, implementation, and evaluation of a pilot prevention model of care within South West Queensland with high unmet needs of chronic disease offers the opportunity to enhance the knowledge of community-based prevention and understand the influence on the uptake of prevention programs. The evaluation will inform the feasibility of Wellness my Way, with findings anticipated to inform potential scale-up via multisector collaborations.

## Abbreviations

AUDRISK: Australian Diabetes Risk Assessment Tool
CRM: customer relations management
ERIC: Expert Recommendations for Implementing Change
HCC: Queensland Health’s Health Contact Centre
HWCRI: Health and Wellbeing Centre for Research Innovation
HWQld: Health and Wellbeing Queensland
NCD: noncommunicable diseases
PHQ-4: 4-item Patient Health Questionnaire
RE-AIM: Reach, Effectiveness, Adoption, Implementation, and Maintenance
SWHHS: Queensland Health South West Hospital Health Service
TIDieR-PHP: Template for Intervention Description and Replication–Population Health and Policy Interventions
UQ: The University of Queensland
